# Comparison of commercial kits to measure cytokine responses to *Plasmodium falciparum *by multiplex microsphere suspension array technology

**DOI:** 10.1186/1475-2875-10-115

**Published:** 2011-05-09

**Authors:** Tamara K Berthoud, Maria Nelia Manaca, Diana Quelhas, Ruth Aguilar, Caterina Guinovart, Laura Puyol, Arnoldo Barbosa, Pedro L Alonso, Carlota Dobaño

**Affiliations:** 1 Barcelona Centre for International Health Research, Hospital Clínic, Universitat de Barcelona, Barcelona, Spain; 2 Centro de Investigação em Saúde da Manhiça, Manhiça, Maputo, Mozambique

## Abstract

**Background:**

Multiplex cytokine profiling systems are useful tools for investigating correlates of protective immunity. Several Luminex and flow cytometry methods are commercially available but there is limited information on the relative performance of different kits. A series of comparison experiments were carried out to determine the most appropriate method for our subsequent studies.

**Methods:**

Two Luminex methods were compared, the Bio-Rad human 17-plex panel and the Invitrogen (formerly BioSource) human cytokine 10-plex kit, and two flow cytometry methods, the Becton Dickinson Human Th1/Th2 Cytokine Kit (CBA) and the Bender MedSystems Human Th1/Th2 11plex FlowCytomix Multiplex Kit. All kits were tested for the measurement of cytokines in supernatants collected from human leukocytes stimulated with viable *Plasmodium falciparum *infected red blood cells (iRBC) or *P. falciparum *schizont lysates.

**Results:**

Data indicated that the kits differed in sensitivity and reproducibility depending on the cytokine, and detected different quantities of some cytokines. The Bio-Rad 17-plex kit was able to detect more positive responses than the Invitrogen 10-plex kit. However, only when detecting IL-1, IL-6 or TNF did the two Luminex based methods correlate with one another. In this study, the flow cytometry based techniques were less variable and correlated better with one another. The two flow cytometry based kits showed significant correlation when detecting IFN-γ, IL-2, TNF, IL-10 and IL-6, but overall the BD kit detected more positive responses than the Bender MedSystems kit.

**Conclusions:**

The microsphere suspension array technologies tested differed in reproducibility and the absolute quantity of cytokine detected. Sample volume, the number of cytokines measured, and the time and cost of the assays also differed. These data provide an accurate assessment of the four techniques, which will allow individual researchers to select the tool most suited for their study population.

## Background

Cells from both the adaptive and innate arms of the immune system secrete small proteins known as cytokines. These proteins are essential to the function of the human immune system. Cytokines perform their actions by binding to cell surface receptors, triggering a signal cascade leading to the modification of gene expression. Thus, the release of cytokines can have direct action on cells. Cytokines can stimulate or inhibit cell activation, they can promote proliferation, direct migration, induce death, and promote cell maturation [1]. Knowledge of the type and quantity of cytokine present during an infection can help us to understand important mechanisms involved in immunity and can give clues as to the type of cells present.

In many diseases, such as HIV/AIDS or malaria, an immune correlate of protection has not yet been identified. Protection may be determined by a number of different parameters that may be present at different moments before exposure and during disease progression. Multiplex cytokine profiling systems enable the quantification of many different proteins within a liquid phase sample and therefore can be a useful tool in identifying correlates of protective immunity and in characterizing disease progression and immune responses. Multiplex techniques can measure many different parameters within a small amount of sample material simultaneously thereby reducing any inter-assay variation, sample material, analysis time, cost and human error. Multiplex systems are especially useful tools in large cohort studies where many samples need to be processed, or when testing scarce material such as samples collected from neonates [2,3].

A number of commercial kits are available. However, limited data have been published comparing the relative performance of these methods; it is not clear what is the most robust and cost-effective choice. The purpose of this study was to compare four different multiplex methods to determine whether there is inherent variation between them and also to determine which kit performs the best in terms of sensitivity and reproducibility for the detection of cytokines produced in the supernatants of human peripheral blood mononuclear cells (PBMC) stimulated with *Plasmodium falciparum *antigens. This paper sets out to describe and evaluate two different methods of multiplex cytokines analysis, luminex and flow cytometry, and to compare the performance of two different manufacturers within each method. The Bio-Rad human 17-plex panel and the BioSource human cytokine 10-plex panel (now supplied by Invitrogen) were tested for the Luminex 100 system. The Becton Dickinson (BD) Human Th1/Th2 Cytometric Bead Array (CBA) Kit and the Bender MedSystems Human Th1/Th2 11plex FlowCytomix Multiplex Kit were tested for the flow cytometry-based method.

Differences in sensitivity and reproducibility were observed depending on the method used and even the cytokine detected. In addition, when selecting the appropriate multiplex cytokine profiling method, other factors were important to consider such as amount of sample needed to run the assay, the number of cytokines measured with each kit, and the time and cost of the assays. This study provides useful information to guide the selection of the appropriate microsphere suspension array technology.

## Methods

### Volunteers

PBMC were collected from malaria naïve donors at the blood bank in Hospital Clinic, Barcelona, Spain (2008), and from healthy adults who had grown up in a malaria endemic area, at the Centro de Investigaçao em Saude da Manhiça (CISM), Manhiça, Mozambique (2005-2006). Written informed consent was obtained from the volunteers. Ethics Committees of Mozambique (reference 28/CNBS/04) and Hospital Clínic, Barcelona, Spain (references 2004/2048, 2008/4376) reviewed and approved the study.

Both malaria naïve and malaria exposed PBMC were tested to be representative of the studies conducted in the laboratory, including adaptive immune responses in exposed individuals and innate immune responses in naïve volunteers. In addition, there were limited cell numbers in exposed individuals and the inclusion of naïve volunteers (subject to less blood volume restrictions) allowed obtaining sufficient amounts of supernatant samples for all the analyses.

### PBMC isolation and stimulation

Both live *P. falciparum *infected trophozoite stage red blood cells (iRBC) and *P. falciparum *schizont extract were used as antigen stimuli. iRBC induce a better response and are the preferred stimulus when logistically possible (e.g. naïve volunteers in Barcelona). In Manhiça, it is not feasible to have continuous production of fresh iRBC from in vitro cultures, therefore, schizont lysates were used for the cell stimulations.

PBMC from the malaria naïve subjects were isolated using a Lymphoprep (Axis Shield) gradient and frozen down in foetal calf serum (FCS) (Invitrogen) with 10% DMSO (Sigma). Before analysis, the PBMC were thawed in complete medium: RPMI 1640 with 10% FCS, 100 IU/ml penicillin, 0.1 mg/ml streptomycin (all Sigma, Poole, UK), and 2 mM l-glutamine (GIBCO/Invitrogen, Paisley, UK) with 25 U/ml Benzonase nuclease (Novagen). Two hundred thousand cells were then stimulated for 24 h, 48 h or 72 h with 600,000 live *P. falciparum *infected trophozoite stage iRBC previously purified using Miltenyi Biotec (CA) Vario MACS CS magnetic separation columns (98% enrichment). The kinetics of the various cytokines tested were not known a priori, therefore, the multiple time points were used to capture the peaks of concentrations for the different cytokines. Following the incubation period, the cells were centrifuged and the supernatant collected and frozen at -80°C.

PBMC from malaria exposed Mozambican volunteers were isolated using a Lymphoprep gradient and resuspended in complete medium. A total of 1.2 million fresh PBMC were stimulated with 20 μl of a *P. falciparum *(3D7 strain) schizont extract corresponding to lysate from 2 million synchronized iRBC. The supernatant was collected following incubation for 48 h or 72 h then frozen at -80°C and shipped to Barcelona for analysis.

### Cytokine multiplex methods

Supernatants were thawed and analysed with the multiplex cytokines detection systems. All methods were carried out using the kits that were commercially available in 2007 and according to the manufacturer instructions. The Invitrogen Human cytokine 10-plex panel and the Bio-Rad experiments were carried out in the presence of a representative from the respective manufacturers. All data were analysed as recommended by the manufacturers.

### Luminex

The Luminex system uses microsphere or bead sets marked with differing ratios of two different fluorophores, conjugated with monoclonal antibodies specific for different cytokines or chemokines. In the assay, once the cytokine of interest has bound, a secondary detection antibody specific for the cytokine or chemokine of interest was added. The beads were read on a Luminex machine (Bioplex-100, Bio-Rad) which has two lasers, one that can identify the bead, and thus the cytokine or chemokine it is specific for, and one that can detect the quantity of the detection agent on the bead, and thus the quantity of the cytokine or chemokine.

A serial dilution of the standards for the standard curve was added to the plate in duplicate. Following the generation of a five-parameter logistic curve, the standard recovery was calculated using the following equation: (observed concentration/expected concentration)*100. A recovery range between 80-120% was recommended by Bio-Rad, and a recovery range between 70-130% was recommended by Invitrogen. Any sample that fell on an area of the curve that was outside these ranges was not considered accurate.

A positive sample was considered if it was above the limits of detection as determined by the manufacturer. The supplementary table shows the mean limits of detection for the Bio-Rad and Invitrogen Kits.

### Flow cytometry

To distinguish bead populations in the flow-based methods, the BD assay uses beads that are stained with different quantities of two fluorochromes, and the Bender MedSystems method distinguishes the beads first by size, then by quantities of a single fluorochrome. Monoclonal antibodies specific to one cytokine or chemokine are conjugated to the surface of the beads. When the cytokine or chemokine of interest has bound, a secondary antibody and detection fluorochromes are conjugated to enable cytokine detection. All samples were acquired and analysed on a FACS Canto II (BD).

The standard curve for the BD CBA assay was determined using a five-parameter logistics model as with the other assays. The data was analysed with FCAP Array software provided by BD. To determine the standard recovery range the following method was used as recommended by BD:

A cut off (CO) value was calculated using the following equation = [a + log MFI(NS)] * b + [c + log MFI(PS)] * d

where: MFI(NS) = the median mean fluorescence intensity of the negative control

MFI(PS) = the median mean fluorescence intensity of the positive control

a, b, c, d are constants (FCAP Array software BD v1.0.1 for windows)

A regression coefficient was then used to determine the fit to the standard curve. The standard curve must have an R-square value of over 98%.

The standard curve and recovery range for the Bender MedSystems assay was determined in the same way as the Invitrogen and Bio-Rad assays outlined above. The recovery range recommended by Bender MedSystems for this assay was between 70-130%. Any sample that fell on an area of the curve that was outside the accepted recovery range was not considered accurate.

A positive sample was considered if it was above the detection limit for each analyte as determined by the manufacturers. The detection limits are listed in the supplementary table.

### Statistical analysis

The values of the cytokine responses were positively skewed, therefore, logarithms were taken of the data prior to analysis. The resulting data were normally distributed. A test of mean differences was performed on the log-transformed data [4]. This involves calculating the difference between the values (between duplicate plates or between different manufacturers for other comparisons), for each sample in turn. The mean (and its 95% confidence interval, CI) of these differences for each sample were calculated. Both of these were then transformed back onto the original scale by anti-logging, to get a ratio change in the raw cytokine responses, which for the mean, is then converted into a percentage change. The percentage differences in cytokine responses were a measure of bias (or systematic differences) between the manufacturers (or two plates). An assessment of whether or not there was some association between measurements by one manufacturer compared to measurements by the other manufacturer (or plate) was done by the Spearman Rank correlation coefficient and its associated p-value; significant negative correlations were deemed implausible and therefore likely to be due to chance. To test if there were significant differences between the amounts of cytokines detected by each assay, a Wilcoxon signed rank test was used.

## Results

### Bio-Rad human 17-plex panel kit

#### Variability between plates

Plate-to-plate variability was tested only with the Bio-Rad human 17-plex panel kit due to limitations in sample volumes. Thirty-two samples from malaria exposed volunteers stimulated with *P. falciparum *schizont lysate for 48 h or 72 h were tested on the same day with the same operators in the presence of a Bio-Rad representative. The number of positive samples detected by each duplicate plate is shown in Table 1. In 14 of the 17 cytokines tested, a difference in the number of positive responses was detected between the duplicate assays.

**Table 1:** Number of positive responses detected by the Bio-Rad Plate 1 and Plate 2

Cytokine	Plate 1	Plate 2
IL-1β	32/32	31/32

IL-2	17/32	12/32

IL-4	26/32	21/32

IL-5	8/32	11/32

IL-6	19/32	32/32

IL-7	3/32	4/32

IL-8	0/32	0/32

IL-10	8/32	30/32

IL-12p70	2/32	2/32

IL-13	26/32	19/32

Il-17	25/32	1/32

G-CSF	30/32	12/32

GM-CSF	0/32	1/32

IFN-γ	19/32	4/32

MCP-1	0/32	0/32

MIP-1β	15/32	31/32

TNF	17/32	9/32

Total All Cytokines	247	220

Concordance between the two plates was investigated in the positive samples. Table 2 gives the Spearman's Rho correlation, the geometric mean (GM) for each plate and the percentage difference in GM (95% CI). The correlation between the two plates differed depending on the cytokine tested. IL-2 and IL-6 showed the best correlation (Spearman's Rho = 0.96 for IL-2 and 0.95 for IL-6, both with P = 0.0001). TNF, IFN-γ and IL-5 did not significantly correlate with one another. Scatter graphs of Plate 1 and Plate 2 for all the cytokines are shown in Additional file 1. The percentage difference in GM between the two plates also varied depending on the cytokine measured. A 162% difference between the two plates was detected in the 15 MIP-1β positive samples even though the two plates significantly correlated with one another. This suggested that plate 1 detected much higher amounts of this chemokine than plate 2. IL-5, IL-13 or IFN-γ showed a lower percentage difference in GM; 14%, 18% and 18% respectively (Table 2). The mean plate-to-plate variability (% difference in GM between duplicates) was 44%.

**Table 2:** Comparisons between two identical plates tested with the Bio-Rad Human 17-plex cytokine panel.

Cytokine	Number of positive observations	Spearman's Rho	GM (pg/ml) plate 1	GM (pg/ml) plate 2	% difference in GM (95% CI) between 2 plates
IL-1 β	31	0.88**	32.47	21.34	52 (32, 75)

IL-2	12	0.96**	30.86	43.76	29 (16, 41)

IL-4	19	0.63**	1.88	1.21	55 (35, 78)

IL-5	7	0.71^NS^	2.16	2.52	14 (-23, 40)

IL-6	19	0.95**	345.36	248.76	38 (20, 60)

IL-10	8	0.86**	21.65	15.06	44 (-19, 155)

IL-13	18	0.86**	5.30	4.49	18 (-8, 52)

G-CSF	12	0.62*	118.08	51.06	131 (92, 179)

IFN-γ	4	-0.60^NS^	85.55	103.95	18 (-50, 55)

MIP-1β	15	0.89**	156.73	59.85	162 (117, 217)

TNF	9	-0.13^NS^	61.10	40.35	51 (2, 126)

Spearman's Rho was used to test if a significant correlation existed between the samples on the two plates. **= Spearman's Rho test with significance of P < 0.01, * = Spearman's Rho test with significance of P < 0.05, ^NS ^= Spearman's Rho test which is non-significant. The cytokines with too few positive observations to enable spearman's Rho tests were not included on this table. GM = geometric mean.

### Bio-Rad human 17-plex panel kit and Invitrogen human cytokine 10-plex panel Comparisons

The sensitivity of the Bio-Rad kit was compared to the Invitrogen kit using 17 supernatant samples from PBMC isolated from Mozambican volunteers and stimulated with *P. falciparum *schizont lysate for 48 h or 72 h. Table 3 shows the number of positive samples detected in Bio-Rad and Invitrogen assays. The Bio-Rad assay detected a greater number of overall positive responses than the Invitrogen assays (88 and 57 respectively). The Invitrogen kit failed to detect any IL-4, IL-5, IL-8 or IFN-γ in any of the samples. The Bio-Rad kit detected at least 1 positive response for all the cytokines apart from IL-8. The Invitrogen kit however did detect a larger number of positive responses than the Bio-Rad kit for GM-CSF and IL-6 despite having a higher limit of detection (according to the manufacturers: Bio-Rad = 2.2 pg/ml GM-CSF, Invitrogen = 15 pg/ml GM-CSF, Bio-Rad = 2.6 pg/ml IL-6 Invitrogen = 3 pg/ml IL-6 as shown in Additional file 2).

**Table 3:** Number of positive responses detected by the Bio-Rad and Invitrogen kits.

Cytokine	Bio-Rad	Invitrogen	Number (%) of concordant positive observations
IL-1β	17/17	17/17	17 (100%)

IL-2	10/17	1/17	1 (5.8%)

IL-4	15/17	0/17	0 (0%)

IL-5	5/17	0/17	0 (0%)

IL-6	7/17	16/17	7 (41.2%)

IL-8	0/17	0/17	0 (0%)

IL-10	9/17	1/17	1 (5.8%)

GM-CSF	1/17	13/17	1 (5.8%)

IFN-γ	12/17	0/17	0 (0%)

TNF	12/17	9/17	9 (52.9%)

TOTAL all cytokines	88	57	

**Figure 1:**
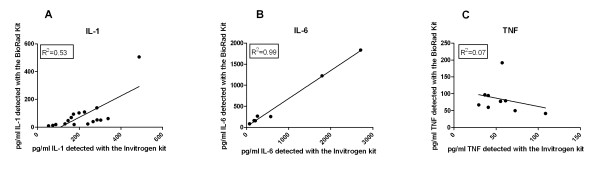
**Correlations between IL-1, IL-6 and TNF measured by Luminex**. Correlation of pg/ml of IL-1 (A), IL-6 (B) and TNF (C) detected between the Bio-Rad and Invitrogen kits. The two kits significantly correlated with one another for the detection of IL-1 (Spearman's Rho test with significance of P < 0.01 = 0.6348) and IL-6 (Spearman's Rho test with significance of P < 0.01 = 0.9286).

A Spearman's Rho test showed that within the three cytokines where enough positive responses were detected to enable statistical comparisons (IL-1, IL-6 and TNF), the two kits significantly correlated with one another in the detection of IL-1 and IL-6 but not TNF (Table 4 and Figure 1). The GM of both IL-1 and IL-6 detected by the Invitrogen assay was higher than the GM for the Bio-Rad assay. A Wilcoxon rank-sum test for significance showed that these differences were significant (Table 4). No significant difference in the quantity of TNF detected by the two kits was seen.

**Table 4:** Comparison of the Bio-Rad and Invitrogen kits.

Cytokine	Number of positive observations	Spearman's Rho	GM (pg/ml) Invitrogen	GM (pg/ml) Bio-Rad	% difference in GM (95% CI)	Wilcoxon Signed Rank Test P value
IL-1β	17	0.6348**	185.86	48.51	283 (174, 436)	0.0004

IL-6	7	0.9286**	408.19	316.03	29 (-3, 73)	0.0008

TNF	9	-0.4017^NS^	52.02	76.48	32 (-60,15)	0.09

Number of positive observation concordant between both assays with the Spearman's Rho to test for significant correlations between the assays. **= Spearman's Rho test with significance of P =< 0.01, * = Spearman's Rho test with significance of P =< 0.05, ^NS ^= Spearman's Rho test which in non-significant. Geometric means (GM) of both assays with the % difference in the geometric means. The table also shows Wilcoxon Rank Sum Significant differences in the quantity of cytokine detected in samples between the Bio-Rad and Invitrogen assays. GM = geometric mean.

### BD CBA and Bender MedSystems FlowCytomix Comparisons

The flow cytometry methods were compared using 73 supernatant samples collected from PBMC stimulated with either *P. falciparum *schizont lysate or iRBC. The number of positive responses detected by each kit is listed in Table 5. The BD kit detected more positive responses than the Bender MedSystems kit overall (204 and 160 respectively) but fewer positive responses for IFN-γ and IL-2. The BD kit detected many more positive responses than the Bender MedSystems kit for IL-10 despite having a higher cut off of sensitivity (BD = 2.8 pg/ml, Bender MedSystems = 1.9 pg/ml as shown in Additional file 2). For IL-2, the Bender MedSystems kit detected more positive samples than the BD kit despite having a higher cut off (BD = 2.6 pg/ml, Bender MedSystems = 16.4 pg/ml as shown in Additional file 2). This suggests that the sensitivity cut off described by the manufacturer does not reflect reality, and is not the reason samples are being detected by one kit, but not the other.

**Table 5:** Number of positive responses detected with the BD and the Bender MedSystems kits.

Cytokine	BD CBA	Bender MedSystems
IFN-γ	23/73	28/73

IL-2	19/73	29/73

IL-4	0/73	0/73

IL-6	73/73	70/73

IL-10	49/73	20/73

IL-12	0/73	1/73

TNF	47/73	44/73

TOTAL all cytokines	204	160

The two flow cytometry methods were compared using 73 supernatant samples collected from PBMC stimulated with either *P. falciparum *schizont lysate or iRBC

**Figure 2:**
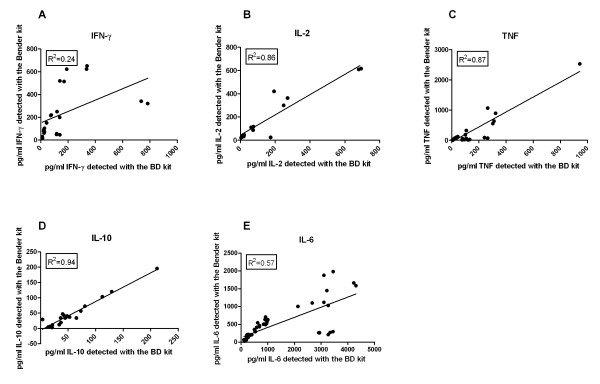
**Correlations between IFN-γ, IL-2, TNF, IL-10 and IL-6 measured by Flow Cytometry**. Scatter plots to show the correlation between the BD and Bender MedSystems kits for IFN-γ (A), IL-2 (B), TNF (C) IL-10 (D), IL-6 (E). The two kits significantly correlated with one another for the detection of all cytokines (Spearman's Rho test with significance of P < 0.01).

All cytokines with enough positive observations to enable a Spearman's Rho (IL-2, IL-6, IL-10, IFN-γ and TNF) showed there was a significant correlation between the BD and the Bender MedSystems (Table 6). However, at least a 29% difference in the GM between the two assays was detected (Table 6). The correlation between the two methods is graphically represented in Figure 2 for IFN-γ, IL-2, IL-6, IL-10, and TNF. The BD kit detected higher quantities of IL-6 and IL-10, whereas the Bender MedSystems kit detected more IFN-γ, IL-2 and TNF (Table 6). A Wilcoxon signed rank test was used to see if these differences were significant. The Bender MedSystems kit detected significantly higher amounts of IFN-γ and TNF but significantly lower amounts of IL-6 and IL-10 than the BD kit. There was no significant difference in IL-2 detection (Table 6).

**Table 6:** Comparison of the BD and Bender MedSystems Kits

Cytokine	Number of positive observations	Spearman's Rho	GM (pg/ml) BD	GM (pg/ml) Bender Medsystems	% difference in GM between 2 plates (95% CI)	Wilcoxon signed rank test
IFN-γ	23	0.75**	80.00	145.69	45 (62, 21)	0.043

IL-2	13	0.86**	80.87	113.10	29 (57, 19)	0.339

IL-6	70	0.94**	867.64	433.89	99.9 (72,133)	<0.0001

IL-10	20	0.88**	40.84	29.87	37 (-5, 96)	0.002

TNF	44	0.7**	36.83	56.64	35 (51, 13)	0.010

Number of positive observation concordant between both assays, with the Spearman's Rho to test for significant correlations between the assays. **= Spearman's Rho test with significance of P < 0.01, * = Spearman's Rho test with significance of P < 0.05, ^NS ^= Spearman's Rho test which in non-significant. Geometric means of both assays with the % difference in geometric means. The table also shows Wilcoxon Rank Sum Significant differences in the quantity of cytokine detected in samples between the BD and Bender MedSystems kits. GM = geometric mean.

### Comparison between duplicate wells - all four methods

Table 7 shows the Spearman's Rho correlation between duplicate wells that were tested within the Bio-Rad, Invitrogen, Bender MedSystems and BD kits. The BD, Bender MedSystems and Bio-Rad assays all appeared to be robust and had good concordance between duplicate wells (Spearman's Rho > 0.8). The Invitrogen assay was more variable with lower concordance between duplicate wells for IFN-γ, IL-2 and TNF.

**Table 7:** Spearman's Rho correlation between the duplicates within the Bio-Rad, Invitrogen, BD and Bender MedSystems kits.

Cytokine	Bio-Rad	Invitrogen	BD	Bender MedSystems
IFN-γ	0.97	0.77	0.99	0.96

IL-2	0.95	0.71	0.99	0.85

IL-6	0.95	0.94	0.94	0.98

IL-10	0.98	0.90	0.89	1

TNF	0.93	0.78	0.96	0.96

IL-4	0.96	0.83	No responses	No Responses

IL-12p70	0.93	Not Done	No responses	No Responses

Mean	0.96	0.95	0.95	0.82

### Comparison of kit characteristics

When selecting an assay to use, other factors are important to consider. Table 8 summarizes the four assays discussed in this paper in terms of cost at the time of the study, number of cytokines detected, amount of sample needed and time for assay completion.

**Table 8:** Summary of the four assays

Measure	Bio-Rad	Invitrogen	BD	Bender MedSystems
Cost (1 = highest cost)	2	1	3	4

Time taken for assay completion	2 h 10 min	5 h	4 h	4 h

Time taken for reading samples	30 min	30 min	2 1/2 h	2 1/2 h

Mean correlation (duplicate wells)	0.95	0.82	0.95	0.96

Number of cytokines tested	17	10	11	7

Volume of sample needed	100 μl	100 μl	50 μl	25 μl

The kits were compared in terms of cost, time for assay completion, correlation between duplicate wells, variability, number of cytokines detected and sample volume. Cost was based on quotes received from Spanish distributers (2007).

## Discussion

Four different multiplex cytokine detection kits from different manufacturers were tested and compared in their ability to detect human chemokines and Th1 and Th2 cytokines in supernatants collected from human PBMC stimulated with *P. falciparum *parasite antigens. A comparison of all parameters across all four methods in parallel with identical sets of supernatants and multiple experiments was not possible because of sample volume restrictions due to limitations in bleeding protocols in the human field studies.

The Bio-Rad human 17-plex Luminex kit was found to be variable even when running the same samples on the same day. TNF, IL-5 and IFN-γ, when detected on duplicate plates, did not significantly correlate. A significant difference in the quantity of some chemokines, such as MIP-1β and G-CSF was also detected between duplicate plates. The second Luminex-based method tested, the Invitrogen 10-plex Luminex kit, was shown to have low concordance between duplicate wells tested on the same plate for some key cytokines: TNF, IL-2 and IFN-γ (Table 7). These results suggest Luminex kits tested in this study might be sensitive to the effect of external factors such as temperature, pipetting and the serum present in the media. Normal human serum has been reported to reduce the sensitivity of some commercial sources of antibody pairs [5], and the presence of animal proteins such as bovine serum albumin can also interfere with the assays. Heterophile antibodies (antibodies able to cross phyla in their reactivity) and human anti-animal antibodies can bind directly to blocking protein causing elevated values, false-positive results and high background [6].

When comparing the Bio-Rad kit to the Invitrogen kit, the Bio-Rad kit was able to detect more positive responses across all cytokines than the Invitrogen kit. A correlation was only seen between the two kits in IL-1 and IL-6 detection. The Invitrogen kit did however detect significantly higher quantities of IL-6 and IL-1 than the Bio-Rad kit. Siawaya *et al *compared three commercial Luminex kits to ELISA assays for the detection of cytokines in whole blood, and reported some differences in accuracy and correlations between different kits. Notably, in their hands, the Bio-Rad human 17-plex assay was shown to correlate less well with ELISA than the other two kits tested (LINCO Inc's 29-plex kit, and RnD System's Fluorokine-Multi Analyte Profiling (MAP) base kit A and B) [7].

The performance of two flow cytometry based kits was also evaluated. The BD Human Th1/Th2 Cytokine kit detected more cytokine-positive samples across all the cytokines, but a significantly lower quantity of IL-6 and IL-1 than the Bender MedSystems Human Th1/Th2 11plex FlowCytomix Multiplex kit. There was good concordance between the two kits when detecting IFN-γ, IL-2, IL-6, IL-10 and TNF. The amount of IL-4 and IL-12 in these samples was low and, therefore, neither kit was able to detect any IL-4 or IL-12. The correlations between the BD kit and Bender MedSystems kit infer that these kits are more robust and less sensitive to external factors than the Luminex-based kits tested.

Significant correlation in IL-6 detection was seen between different kits with both the Luminex and flow cytometry-based methods. These correlations could be due to the high levels of IL-6 detected by all assays insuring that the levels were well above the limits of detection and the quantities more accurately determined. Khan *et. al. *compared four Luminex-based methods for the detection of cytokines in serum and also reported that the trends and concentrations of IL-6 detected did not 'differ substantially' across the kits, the same was observed for IL-8 and TNF [8]. It is possible that IL-6 antibodies are less affected by external factors and bind more efficiently than other antibodies.

Differences in the absolute quantity of cytokine present in the samples were detected when testing both the Luminex and the flow cytometry systems. This has been reported elsewhere with different commercially available ELISA and multiplex kits [9-11]. Young *et al *compared the BD kit, ELISA and Bender MedSystems kit in the measurement of cytokines in rodent bronchoalveolar lavage and reported that even though correlations can be good between multiplex cytokine kits and ELISA, a large fold difference (up to 11-fold) in the quantity of cytokine detected can be seen [11]. Contaminants in the matrix that differentially affect the standard curves and efficiency of different monoclonal antibody pairing may account for these differences.

These results have highlighted the differences that may exist between multiplex microsphere suspension array cytokine kits. It is important to be aware of the potential differences in absolute cytokine measurements between kits when comparing different samples tested with different methods. The large variation seen between duplicate plates (Bio-Rad) may require that rigorous standards be in place to prove that data between plates are indeed comparable. Other factors such as cost, quantity of sample needed and number of parameters measured are also important to consider. In addition, sensitivity for each cytokine varies within a multiplex kit and the final selection may be influenced by which cytokines are of more interest. The Luminex assays offered a greater variety of cytokines than flow-based methods, but in this study were more expensive and more variable with the kits available at the time. Only two of the several Luminex kits available for multiplex cytokine analysis were tested, these kits are evolving and improvements can be introduced. In the most recent Luminex kits, availability of magnetic beads may decrease bead loss during washes and increase the reproducibility and robustness of the new Luminex-based kits.

## Conclusions

Profiling systems are invaluable when there is a need to screen many samples or cytokines, or when much information is needed from a small amount of sample. These data provide an accurate assessment of the four techniques, which will allow individual researchers to select the tool most suited for their malaria studies. It is important to be aware of the potential differences between the manufacturers before selecting a kit and when comparing cytokine concentrations detected with two different commercial kits. If more precise measurement of the amount of a particular cytokine present within a sample is needed, probably a different method such as super-sensitive ELISA should be considered.

## Competing interests

The authors declare that they have no competing interests.

## Authors' contributions

CG and PLA carried out the human field studies. MNM, DQ, RA, AB processed the blood samples and carried out the antigen stimulations in Manhiça. TKB and LP carried out the immunoassays in Barcelona. CD and TKB conceived and designed the study, acquired, analysed and interpreted the data, and wrote the first draft of the paper. All authors read and approved the final manuscript.

## Supplementary Material

Additional file 1 **Correlations between Bio-Rad plate one and plate two**. Scatter plots to show the correlations between IL-1, IL-2, IL-4, IL-5, IL-6, IL-7, IL-17, IL-10, IL-12, IL-13, G-CSF, IFN-γ and TNF on two duplicate plates, run on the same day with the same samples. Click here for file

Additional file 2 **Supplementary Table: Kit Detection Limits**. Detection limits (pg/ml) provided by manufacturers for cytokines detected in the Bio-Rad human 17-plex panel, the Invitrogen human cytokine 10-plex panel, the BD human Cytometric Bead Array (CBA) Human Th1/Th2 Cytokine Kit and the Bender MedSystems Human Th1/Th2 11plex FlowCytomix Multiplex Kit. Cytokines not included in a particular kit are marked 'Not tested'. Click here for file
