# Supplementary material for: Comparison of commercial kits to measure cytokine responses to Plasmodium falciparum by multiplex microsphere suspension array technology
Source: Malar J. 2011 May 9;10:115. doi: 10.1186/1475-2875-10-115 (PMC3112452; doi:10.1186/1475-2875-10-115)
Supplement: Additional file 2 — Supplementary Table: Kit Detection Limits. Detection limits (pg/ml) provided by manufacturers for cytokines detected in the Bio-Rad human 17-plex panel, the Invitrogen human cytokine 10-plex panel, the BD human Cytometric Bead Array (CBA) Human Th1/Th2 Cytokine Kit and the Bender MedSystems Human Th1/Th2 11plex FlowCytomix Multiplex Kit. Cytokines not included in a particular kit are marked 'Not tested'. [file 1475-2875-10-115-S2.RTF]

Additional file 2  - Supplementary Table: Kit Detection Limits 
Cytokine (Human)	Bio-Rad (pg/ml)	Invitrogen (pg/ml)	BD             (pg/ml)	Bender MedSystems (pg/ml)	
IFN-ã	6.4	5	7.1	1.6	
IL-1â	0.6	15	Not tested	4.2	
IL-2	1.6	6	2.6	16.4	
IL-4	0.7	5	2.6	20.8	
IL-5	0.6	3	Not tested	1.6	
IL-6	2.6	3	3.0	1.2	
IL-7	1.1	Not tested	Not tested	Not tested	
IL-8	1.0	3	Not tested	0.5	
IL-10	0.3	5	2.8	1.9	
IL-12p70	3.5	Not tested	1.9	1.5	
IL-13	0.7	Not tested	Not tested	Not tested	
IL-17	3.3	Not tested	Not tested	Not tested	
G-CSF	1.7	Not tested	Not tested	Not tested	
GM-CSF	2.2	15	Not tested	Not tested	
MCP-1	1.1	Not tested	Not tested	Not tested	
MIP-1â	2.4	Not tested	Not tested	Not tested	
TNF	6.0	10	2.8	3.2	
TNF-â	Not tested	Not tested	Not tested	2.4	
